# Laparoscopic versus robotic-assisted sacrocolpopexy for pelvic organ prolapse: a systematic review

**DOI:** 10.1007/s10397-016-0930-z

**Published:** 2016-01-26

**Authors:** Geertje Callewaert, Jan Bosteels, Susanne Housmans, Jasper Verguts, Ben Van Cleynenbreugel, Frank Van der Aa, Dirk De Ridder, Ignace Vergote, Jan Deprest

**Affiliations:** Department of Development and Regeneration, Cluster Organ Systems, Faculty of Medicine, Group Biomedical Sciences, KU Leuven, 3000 Leuven, Belgium; Department of Obstetrics and Gynaecology, University Hospitals Leuven, 3000 Leuven, Belgium; Belgian Center for Evidence Based Medicine (CEBAM), Belgian Branch of the Cochrane Collaboration, 3000 Leuven, Belgium; Department of Obstetrics and Gynaecology, Jessa Hospital, 3500 Hasselt, Belgium; Department of Urology, University Hospitals Leuven, Leuven, Belgium; Department of Gynaecologic Oncology, Leuven Cancer Institute, University Hospitals Leuven, KU Leuven, 3000 Leuven, Belgium

**Keywords:** Sacrocolpopexy, Laparoscopy, Pelvic organ prolapse, Vault prolapse, Robotics, Costs

## Abstract

The use of robot-assisted surgery (RAS) has gained popularity in the field of gynaecology, including pelvic floor surgery. To assess the benefits of RAS, we conducted a systematic review of randomized controlled trials comparing laparoscopic and robotic-assisted sacrocolpopexy. The Cochrane Library (1970–January 2015), MEDLINE (1966 to January 2015), and EMBASE (1974 to January 2015) were searched, as well as ClinicalTrials.gov and the International Clinical Trials Registry Platform. We identified two randomized trials (*n* = 78) comparing laparoscopic with robotic sacrocolpopexy. The Paraiso 2011 study showed that laparoscopic was faster than robotic sacrocolpopexy (199 ± 46 vs. 265 ± 50 min; *p* < .001), yet in the ACCESS trial, no difference was present (225 ± 62.3 vs. 246.5 ± 51.3 min; *p* = .110). Costs for using the robot were significantly higher in both studies, however, in the ACCESS trial, only when purchase and maintenance of the robot was included (LSC US$11,573 ± 3191 vs. RASC US$19,616 ± 3135; *p* < .001). In the Paraiso study, RASC was more expensive even without considering those costs (LSC US$ 14,342 ± 2941 vs. RASC 16,278 ± 3326; *p* = 0.008). Pain was reportedly higher after RASC, although at different time points after the operation. There were no differences in anatomical outcomes, pelvic floor function, and quality of life. The experience with RASC was tenfold lower than that with LSC in both studies. The heterogeneity between the two studies precluded a meta-analysis. Based on small randomized studies, with surgeons less experienced in RAS than in laparoscopic surgery, robotic surgery significantly increases the cost of a laparoscopic sacrocolpopexy. RASC would be more sustainable if its costs would be lower. Though RASC may have other benefits, such as reduction of the learning curve and increased ergonomics or dexterity, these remain to be demonstrated.

**Electronic supplementary material:**

The online version of this article (doi:10.1007/s10397-016-0930-z) contains supplementary material, which is available to authorized users.

## Introduction

### Robotic-assisted surgery

Robot-assisted surgery (RAS) has become popular in various surgical fields, including gynaecology and urology. Accordingly, the robot has been used for the surgical treatment of pelvic organ prolapse (POP). The most frequently quoted advantages of RAS are its 3D view, the elimination of surgeon tremor whilst permitting precise and intuitive movements. Further to this the use of wristed instruments improves dexterity offering more favorable ergonomics. RAS combines these advantages with the minimally invasive approach, i.e., those already demonstrated for conventional or “straight stick” laparoscopy. Wide introduction of RAS is mainly limited by the high acquisition and maintenance cost (usually around 10 % of the purchase cost per year) and the repetitive costs of the consumables [1]. In Europe, the *initial cost* is typically depreciated over 7 or more years, which amounts to more than 1000 € per patient, when used for 300 or more procedures per year [2]. When used in fewer patients, this will result in higher per-case charges. The use of robotic instruments is limited to 10 cases, and the list charge price for three instruments is easily more than 1500 € [3]. Next to the high costs, other disadvantages are the lack of tactile feedback and instrument crowding, especially in a narrow operating field, such as the pelvis [2].

### Minimally invasive pelvic floor surgery

Whereas most patients with symptomatic POP can be adequately managed by the vaginal route, correction of apical descent or multi-compartment prolapse with a so-called level I defect is better treated by the abdominal approach [4]. In sacrocolpopexy (SC), the vaginal vault and/or cervix is fixed by means of a graft to the anterior longitudinal ligament over the sacrum. Sacrocolpopexy by laparotomy further referred to as abdominal sacrocolpopexy (ASC) yields an over 90 % success rate, which improves on sacrospinous fixation. This is however at the expense of longer operation times, higher morbidity, and increased hospital cost [4]. These shortcomings are avoided by performing SC by minimal access, either by laparoscopy (LSC) or by its robotic-assisted equivalent (RASC). Despite the lower performance of spinofixation, single incision vaginal mesh prolapse repair seemed to be a reasonable alternative to LSC, as it was supposed to combine the durability and comprehensiveness of a mesh repair and the advantages of the vaginal route. In Maher’s randomized clinical trial (RCT), LSC was associated with a shorter hospital stay, earlier return to daily activity, better 2-year anatomical outcomes, less graft related complications, and, as a consequence, less reinterventions as well as lower hospital costs, despite longer operation times [5, 6].

Only by 2012, level I evidence became available supporting the hypothesis that a laparoscopic SC yields as good anatomic (point C) and subjective (patient global impression score) outcomes as the same operation by laparotomy [7]. Moreover, LSC was associated with less blood loss, less pain, and a shorter hospital stay. Conversely, operation time, return to normal activities, or functional effects were similar for both modalities.

LSC unfortunately did not become widely implemented, because of its steep learning curve and long operation times, adding to the generic disadvantages of a limited number of degrees of freedom and its two-dimensional vision [8]. These disadvantages could be circumvented by robotic assistance. The da Vinci Surgical System^®^ (Intuitive Surgical Inc., Sunnyvale, CA, USA) is at present the only operational and commercially available surgical robot. Its increased magnification, three-dimensional vision, physiologic tremor filtering, and 7 degrees of freedom are believed to provide the surgeon with an enhanced ergonomic environment, simplifying complex laparoscopic tasks such as suturing and knot tying, which are essential techniques for SC. The implementation of robots was surprisingly quick into the clinical practice of gynaecologists in many Western countries. This may be by a combination of extensive marketing but certainly because RAS answers the needs of some robotic surgeons *not familiar with conventional laparoscopic surgery*. Subsequently, there has been a significant body of reassuring studies on RASCP demonstrating safety and efficacy, reviewed by Serati et al. [9]. Though there is to our knowledge no RCT comparing RASCP to ASCP, it seems that anatomical and functional outcomes are comparable, though with reduced morbidity, which logically would be the consequence of the minimal access route.

This experience has led to statements that RAS is “better than conventional surgery”—which is clearly stated on  the manufacturer’s website [10]. The latter is misleading at least, because today conventional sacrocolpopexy no longer is the synonym of ASC [10]. Given the level I evidence that LSC overall is better than ASC, the laparoscopic approach should theoretically be the standard and point of reference. Herein, we aimed to investigate whether there is at present any evidence that RASC would by any outcome measure be superior to LSC. Acceptable advantages would be respectively, a clinical benefit to the patient, a reduced health care cost, or improved surgeon’s ergonomics. This question is timely: with the aging and increasing activity of the population, the demand for prolapse surgery is only expected to increase. Given the movement away from vaginal mesh use, minimal access sacrocolpopexy will become an increasingly popular procedure.

## Methods

### Literature search strategy

Relevant studies were identified from the Cochrane Library (1970–January 2015), MEDLINE (1966 to January 2015), and EMBASE (1974 to January 2015). Furthermore, ClinicalTrials.gov and the International Clinical Trials Registry Platform were searched for ongoing and completed clinical trials. Language restrictions were not applied. There was no systematic attempt to search the grey literature. Details of the search strategy can be found as an online resource. Essentially, RCTs or controlled studies were included if they compared laparoscopic (LASC) with robotic-assisted sacrocolpopexy (RASC) as the primary surgical intervention with or without concomitant surgery.

### Data collection and analysis

All titles and abstracts retrieved by electronic searching were independently assessed by two review authors (GC and JB). Studies that did not meet the inclusion criteria were excluded, and full-text copies of the potential eligible studies were obtained. The full-text articles were assessed for eligibility independently. Disagreements were resolved through discussion or arbitration by a third review author (JD).

For data extraction, a standard form available from the website of the Cochrane Library was used (data collection form for intervention reviews: RCTs only, version 3, April 2014). Two review authors (JB, GC) extracted data from eligible studies. When studies included data from multiple publications, the main trial report was used, supplemented by a previous published protocol if available. Differences were resolved through discussion or arbitration by a third review author (JD).

Primary outcomes were the use of resources and costs (including equipment/theatre costs, length of hospital stay in days, duration of operation in minutes, number of outpatient attendances, number of days off work, direct medical resource use, direct medical costs. Secondary outcomes were patient satisfaction parameters, measured by any validated questionnaire (e.g., PGI-I, PGIC, POP-specific quality of life (P-QoL)); objective measurement of cure rate (POP-Q stage); any complication, either intraoperative, postoperative within 6 weeks, or at a later stage during follow-up, and its nature; early mortality (death within 30 days); estimated blood loss; rate of conversion to open surgery (for RAS versus CLS) and the reason for conversion; and postoperative pain (VAS or other validated scale).

The selected studies were assessed for methodological quality using the “risk of bias” tool of the Cochrane Collaboration [11]. The method of randomization, allocation concealment, blinding, loss to follow-up, selective outcome reporting, trial funding, and if present other sources of bias were again independently evaluated arbitrated as above.

### Statistical analysis

For the main outcome of interest, “use of resources and costs,” the mean values, and the standard deviations (SD) were extracted. For the other outcomes, if binary, the number of events was noted. Since the number of RCTs was limited and the used definitions of essential outcome measures were different, additional meta-analysis was not performed.

## Results

**Fig 1:**
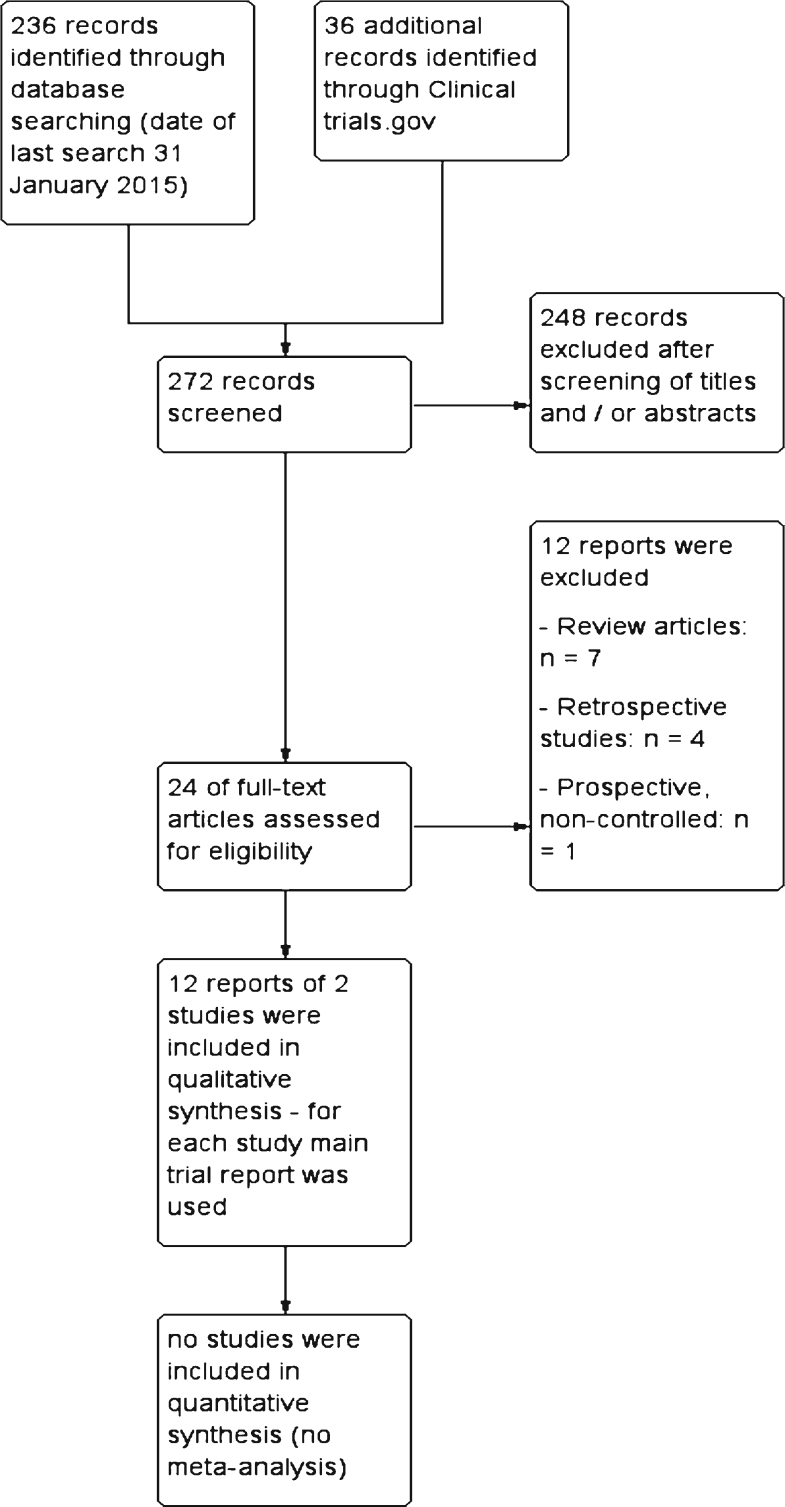
Study flow diagram

A flow diagram of the search process is displayed in Fig. 1. The search yielded 272 citations of which 24 underwent full text review after screening of the titles and abstracts. Twelve manuscripts concerning the two same studies were included in the SR after consideration of the full text. Both studies were RCTs comparing laparoscopic to robotic sacrocolpopexy of similar size (*n* = 78; Anger 2014 and Paraiso 2011); the former had hospital cost as a primary outcome measure, whereas in the latter it was operation time. Both studies reported both variables. The characteristics of these studies are summarized in Table 1; the outcomes in Table 2. Required surgeon’s experience with RASC prior to the study was similar: surgeons were to have performed at least 10 prior robotic procedures. The actual performance was not mentioned in either report. Sacrocolpopexy was performed using two separate pieces of polypropylene mesh. Concomitant procedures were allowed and equally distributed between the two arms in both studies, including retropubic mid-urethral sling and anterior or posterior repairs. Of note is that in Anger’s ACCESS trial, 58 % of women underwent a concomitant hysterectomy, whereas in the study by Paraiso only patients with posthysterectomy vault prolapse were included. Other differences at baseline were the setup (single versus two centers) and indication (vault prolapse and/or uterine descent).

**Table 1:** Study characteristics

Study	Paraiso 2011	Anger 2014
Design	Parallel-group, single-center trial RCT	Two-center, parallel-group RCT
Ethical approval	Yes	Yes
Power calculation	Yes—to detect a 50-min difference in operating time with 90 % power and 5 % type 1 error	Yes—to detect at least US$2500 difference in total charges with 95 % power and 5 % type 1 error
CONSORT statement	Yes	No
Conflict of interest	No conflicts of interest	No conflicts of interest
Participants	Country: USA Setting: Cleveland Clinic Population: women >21 years presenting with posthysterectomy vaginal apex prolapse with POP-Q stage 2–4 desiring surgical management between January 2007 to December 2009 Patients were excluded if not candidates for general anesthesia, underwent a prior sacral colpopexy or rectopexy, had a history of PID, had a BMI >40 kg/m^2^	Country: USA Setting: University of California-Los Angeles/Cedars-Sinai and Loyola University Medical Centers Population: women with symptomatic pelvic organ prolapse stage II or greater and clinical indication for sacrocolpopexy Patients were excluded if future childbearing, pregnant or pregnancy in the last 12 months, unable to read, write, and comprehend English
Interventions	Sacrocolpopexy using 2 separate 4 × 15 cm pieces of polypropylene mesh. Use of 4 ports for the laparoscopy, 5 for the robotic-assisted laparoscopy in W formation	Sacrocolpopexy with 2 separate pieces of polypropylene mesh and Gore-Tex sutures—surgeon’s preference determined brand of the mesh and closure of the retroperitoneal lining
Randomization method	Computer-generated randomization schedule—stratified by surgeon	Computer-based block randomization based on site and need for concurrent hysterectomy randomization on the day of the surgery
Allocation concealment	Use of opaque envelopes	Treatment allocation is uploaded on a password protected website—randomization assignment is revealed to treating surgeon on the day of surgery Under procedure in patient file: laparoscopic sacrocolpopexy per the ACCESS protocol
Blinding	Blinding of research staff and patients	Blinding of patients and research staff for 6 weeks after surgery
Groups comparable	Yes	Yes
Intention-to-treat analysis	Yes	Yes
Follow-up	Up to 1 year	Up to 1 year
Loss to follow-up	4 lost to FU after surgery from LASC 2 from RASC	3/78 before 6 M FU visit
Intervention group	Robotic-assisted sacrocolpopexy (randomized: *n* = 40—underwent surgery *n* = 35)	RASC (*n* = 40)
Control group	Laparoscopic sacrocolpopexy (randomized: *n* = 38—underwent surgery: *n* = 33)	LASC (*n* = 38)
Concomitant surgery	Yes	Yes
Surgical experience	At least 10 robotic procedures	At least 10 procedures of each type
Outcome measures	Primary outcome: operating time Secondary outcomes: postoperative pain, use of NSAIDs, complications, costs, postoperative subjective and objective cure rate	Primary outcome: costs Secondary outcomes: surgical outcomes (blood loss and postoperative pain), POP-Q, symptom severity and QoL, adverse events

**Table 2:** Outcomes

Outcome	Paraiso 2011				Anger 2014		
	LSC (*n* = 33)	RASC (*n* = 35)	Mean difference	P	LSC (*n* = 38)	RASC (*n* = 40)	*P*
Time—sacrocolpopexy	162 ± 47 min	227 ± 47 min	67 (CI 43–89)	<.001	178.4 ± 49.8 min	202.8 ± 46.1 min	.030
Time—total operation operating	199 ± 46 min	265 ± 50 min	66 (43–90)	<.001	225.5 ± 62.3 min	246.5 ± 51.3 min	.110
Costs - Day of surgery - Excluding robotics	° US$14,342 ± 2941	° US$16,278 ± 3326	° US$1936 (448–2,885)	° .008	US$11,573 ± 3191 US$11,573 ± 3191	US$19,616 ± 3135 US$12,586 ± 3315	<.001 .160
Costs - At 6 weeks - Excluding robotics	° °	° °	° °	° °	US$12,170 ± 4129 US$12,170 ± 4129	US$20,898 ± 3386 US$13,867 ± 3386	<.001 .060
Pain - Use of NSAIDs (days) - VAS at nl activity(wk1) - Activity scales (1 wk)	11 days^a^ 28 (2–67)	20 days^a^ 28 (4–68)		<.005^a^ .41	° 2.6 ± 2.2 38.1 ± 15.5	° 3.5 ± 2.1 45.4 ± 16.1	° .044 .039

*LSC* laparoscopic sacrocolpopexy, *RASC* robotic-assisted sacrocolpopexy, *NSAIDs* nonsteroidal anti-inflammatory drugs, *VAS* visual analog scale, *no* data available, *Excluding robotics* excluding costs of purchase and maintenance costs for robot

^a^Only visual scale (no raw data)

**Fig 2:**
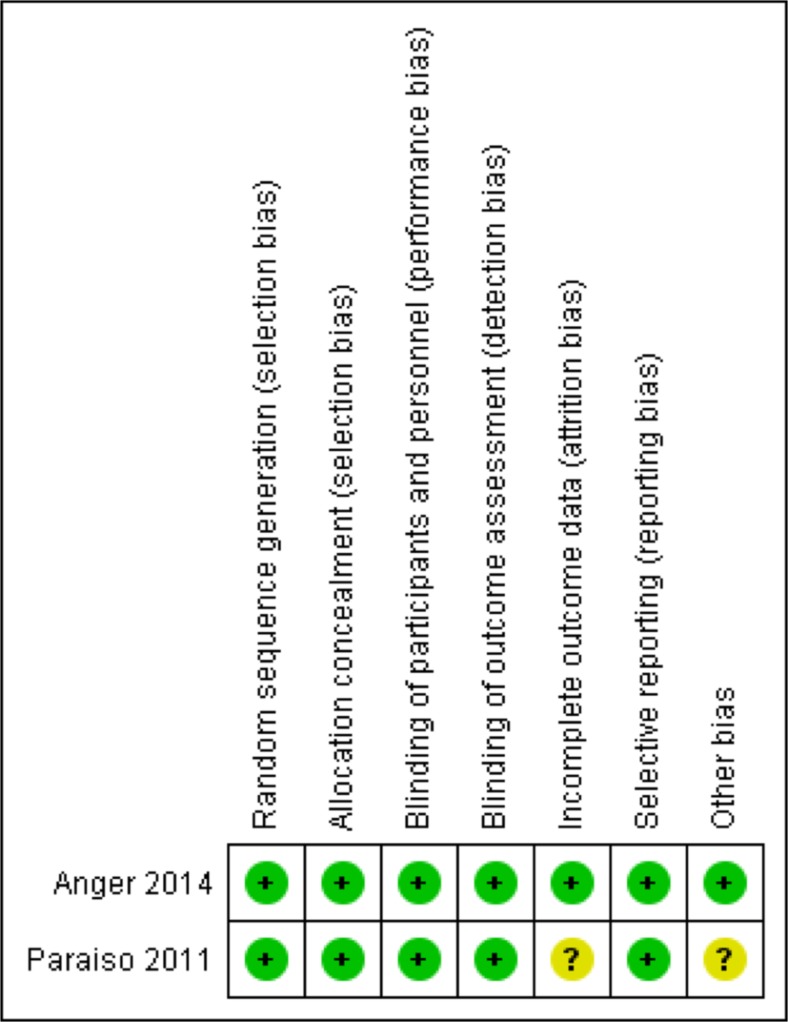
Risk of bias summary: review authors’ judgements about each risk of bias item for each included study

The studies also had comparable secondary outcomes which were limited to surgical complications and blood loss, postoperative pain, objective cure rate, and patient satisfaction. Both studies used a blinded computer-based randomization system, hence having low risk for selection bias. Both assessors and patients were blinded to treatment allocation. The data were analyzed on an intention-to-treat basis. In the study by Paraiso, 13 % of the study population (*n* = 10) was lost after randomization and prior to surgery. Surprisingly, four patients were excluded because they did not meet the criteria, and six others because of patient choice or illness, so attrition bias was considered unclear. Conversely, the attrition bias of the ACCESS trial was low: there were five patients lost to follow-up after the surgery, equally distributed over both treatment arms. The summary of the risk of bias assessment can be found in Fig. 2. Reporting bias is low for both studies; both protocols were also published upfront [12, 13]. Remarkably, the published protocol of the ACCESS trial mentioned initially a follow-up of 1 year postoperatively, yet in the publication a 6-month follow-up is reported on.

Both studies came to different conclusions in terms of the measures initially put forward as primary outcome by either group. In the hands of Paraiso et al., LSC was faster than RASC (199 ± 46 vs. 265 ± 50 min; *p* < .001), yet Anger et al. did not find a difference (225 ± 62.3 vs. 246.5 ± 51.3 min; *p* = .110). Both trials report a significantly higher cost for RASC, yet they use different definitions. The ACCESS breaks down the healthcare costs as those made by the health care provider, first related to the initial procedure, and secondly those related to potential readmissions within 6 weeks. For the surgery-related cost, they report both the purchase of the robot and the maintenance cost as well as its consumables. Eventually, the higher costs in the ACCESS trial (LSC US$11,573 ± 3191 vs. RASC US$19,616 ± 3135; *p* < .001) were only due to the higher purchase and maintenance cost of the robot. Excluding those costs, the healthcare cost was actually comparable, with or without concomitant surgery (LSC US$11,573 ± 3,191 vs. 12,586 ± 3315; *p* = .160) [14]. This is different for the other study. Firstly, Paraiso does not consider the purchase and maintenance cost of the robot. Even when leaving this contributor to costs, they still find significant higher costs for the RASC (LSC US$14,342 ± 2941 vs. RASC 16,278 ± 3326; *p* = 0.008). That difference was primarily driven by the higher operating room cost for RASC, and not the costs related to “hospitalization” [12].

In essence therefore, when using similar definitions of operating cost, there were no differences in costs in one but not in the other study. In a nutshell, the operational costs excluding purchase and maintenance were comparable in the former study, but not in the latter. When it comes to investments, purchase costs were not included in one study [12], though were higher when taken into account. The magnitude is as given above, though it is worth noting that the costs of the laparoscopic equipment was not computed by Anger et al., therefore the difference may be exaggerated.

Clinical outcomes were also reported with no difference in complications. In Paraiso et al., three conversions were reported, all in the robotic arm, one to laparotomy, two to LSC. In Anger et al., there were no conversions. Both studies report higher postoperative pain for RASC patients. This persists for only the first week following surgery in the Anger cohort and between three and five weeks for Paraiso, though the scales used were again not comparable. However supporting this observation in Paraiso's study was the extended use of painkillers following RASC (11 days versus 20 days; *p* < .005) [12]. This was not reported by Anger. Efficacy (anatomical outcome) of the procedure was equivalent on short term (6 months [14] or 1 year [12]). The pelvic floor dysfunction and quality of life questionnaires partially overlapped for both studies, yet there were no differences observed again at slightly different time points.

Because of the differences in definitions of essential (primary) outcome measures, we decided not to pool the results for a formal meta-analysis.

## Discussion

### RASC may be equally effective yet is more expensive

Though efficacy was not a primary outcome in this study, it is reasonable to assume that RASC and LSC are equally efficacious. In reality, it may numerically be very difficult to design a study that would show the opposite. The available randomized trials logically looked at other aspects; one of these being costs. When taking into account the purchase and maintenance costs, the ACCESS trial demonstrates an increased cost when the robot is used [14]. Although the costs of consumables were only reported in one study [12], they are higher than for straight stick LSC. The above is in line with earlier observational studies on the same procedure [15, 16]. Given that hardware and consumable costs are among the principal cost drivers, another result would have been surprising. Other firm contributors are typically operation time and hospital stay, yet they are comparable in one RCT [14] and several observational studies [17, 18] and hence cannot compensate for the increased “material” costs. This is also in line with randomized studies on other procedures, such as hysterectomy [19], fundoplication [20], and right hemicolectomy [21], as well as the numerous observational studies [22–25]. Obviously, one cannot compare all these studies of different design and quality, and it seems that, roughly spoken, one looks at an excess cost of US$2–3000 [26, 27]. Whether one would come to the same magnitude of excess costs in a European setting or elsewhere remains to be studied, yet, proportionally spoken, it is unlikely that there is a cost model where this difference would not show.

Both studies consistently report more short-term pain, though at later timepoints it is difficult to compare outcomes as different measurement methods were used. This aspect has not been very well studied in observational studies on SC, with one study showing similar levels of postoperative pain [17] or (randomized) studies on other procedures. Park et al. found no significant difference in postoperative pain in a RCT comparing robotic and laparoscopic right hemicolectomy in cancer patients [21]. It is therefore difficult to draw a firm conclusion, though the clinical relevance of increased pain sensation over a very short time with limited increase in pain relief is probably not very important. Further research into the actual cause of a difference in pain may demonstrate the blame does not lay with the robot. For instance, it has been speculated that this may be due to an increased diameter and/or increased tension on the robotic ports, which theoretically can be remediated by further technologic advancements.

The question is how solid one considers the evidence for the above conclusions. First, there are the methodological differences on definition of costs, pain, etc. This is inherent when there are no gold standard methods for this type of research. The two available studies may however be individually criticized as well. One can argue what is the most relevant endpoint to study, but it seems completely acceptable to us to take *cost* as a relevant outcome measure in the current economic situation. Whether the choice for a hospital cost analysis rather than a cost study that looks further than that, is right, may be another point of discussion. It seems however fair to us for a hospital to first do a cost-minimization study as management decisions will be primarily based on the outcomes of such study. *Operation time* is also an acceptable endpoint as this proxy for surgical efficiency bears relevance both to surgeons as well as hospital management. From a methodological viewpoint, it could be argued that it is uncertain whether the surgical skills and experience at the onset of the RCT were comparable for both treatment modalities. Both studies state that a minimum of 10 RASC was required, though actual numbers are lacking. We requested that information, and both authors kindly provided their best estimate, ranging between 10 and15 for two surgeons and 50 for two other surgeons for the study by Anger et al. For the study by Paraiso et al., one surgeon had a previous experience of 400–500 LSCs and 10 RASCs and the other surgeon had performed about 100 LSCs and 10 RASCs. Nevertheless, even under the “best” circumstances of a very experienced team, it seems not possible to overcome the limitation of an increased cost.

In summary, these two RCTs are the best available evidence and consistently demonstrate higher costs without measurable benefit to the patient. We therefore agree with Steege and Einarsson [10] that stating “robotic is better than conventional surgery” is not supported by high-quality evidence at present. In the absence of any evidence of superiority, is the “problem of how to implement the technology … moot”, as the Editorial suggests [10]?

### Speculations on future place of robotic in pelvic floor surgery

Nonetheless, this does not mean the robot does not have some assets. Laparoscopic surgeons frequently report discomfort in the neck and upper extremities as well as experience higher stress levels [28–30]. A more static posture during LSC as opposed to open surgery is blamed for this adverse effect [31]. For relatively lengthy operations such as SC (sacrocolpopexy), this may not be a trivial observation. Regardless, this adds to the inherent limited degree of freedom of motion by “straight stick” laparoscopic instruments, which impacts female surgeons more than male and surgeons with smaller hands [30, 32]. Also, in some outdated operating theatres, the inappropriate positioning or limited numbers of monitors may be disturbing [33, 34].

Previously, Tarr et al. studied the ergonomic impact specifically for *sacrocolpopexy* in a prospective cohort of 33 RASC and 53 LSC procedures over a 16-month period [35]. The procedures were performed by a variety of surgeons at different seniority (resident, fellow, attending), though all were previously trained with the studied modalities. As outcome measures, they used a validated five-step score for measuring “Body Part Discomfort” (BPD) in different body regions and the National Aeronautics and Space Administration Task Load Index (NASA-TLI), rating, e.g., mental and physical demands, and effort and frustration with given tasks on a continuous scale. [36]. In a nutshell, this study showed that RASC was associated with lower neck, shoulder, and back discomfort scores [35]. Stress levels, measured by skin conductance level, and heart rate were significantly lower in surgically inexperienced medical students performing tasks with the robot when compared to laparoscopic instrumentation [37]. These findings will need to be validated in a proper RCT and a more homogenous population of surgeons.

Another view is that RASC in the hands of a novice, or of a lesser experienced laparoscopist, is the most safe and effective tool to perform advanced procedures [38]; hence, the economical disadvantages can be ignored. Assuming that one can unequivocally demonstrate that RASC is much easier to be learned than LSC (no study has shown this to our knowledge), it may be, at a larger scale, the most pragmatic solution for the long and demanding learning process typical for this procedure [8, 39, 40]. Indeed, we have shown that 30 LSCs are required to achieve an operation time within the range of an experienced surgeon, and 60 procedures to obtain similar complication rates [39]. This, combined with the relative low numbers of sacrocolpopexies at each individual training unit, makes adequate training problematic [8]. Robotics in a setting with a double console could be safer and more effective to train junior surgeons. This is however far from certain: one study demonstrated that robotic hysterectomy actually had a longer learning curve (*n* = 91) than what we described for LSC [41]. From a training perspective, one could also follow another strategy and try to shorten the learning process, for instance, by a wider introduction of 3-D LSC [42]. This may require an investment—which is probably less than for a robot—yet will not have the repetitive instrument cost.

For us, there is no doubt that robotic developments boost surgical capacities. Articulation beyond normal manipulation, tremor reduction, and all the other claimed advantages may not be proven; however, these properties, and the improved ergonomics, are a true paradigm shift in surgery. For that alone, the technology warrants further development. Certainly in the field of urogynaecology, for some relatively complex procedures, such as mesh removal, or more novel procedures such as those for urge incontinence, which require bilateral extensive retroperitoneal dissections, there might be a benefit [43].

In conclusion, robotic surgery significantly adds to the costs. To make it sustainable, and to allow further investments in a technology which has not reached its limits, we must move to a more reasonable cost. This can either result from the arrival of a competitor (long expected but not yet a reality) [2] or by negotiating a more reasonable and affordable price either for the hospital [44] or at a higher level. The latter  approach has been successfully undertaken in Belgium through negotiation with the medical drug industry [45].

## Electronic supplementary material

Below is the link to the electronic supplementary material.

ESM 1 (PDF 191 kb)
