# Supplementary material for: Laparoscopic versus robotic-assisted sacrocolpopexy for pelvic organ prolapse: a systematic review
Source: Gynecol Surg. 2016 Jan 26;13:115–23. doi: 10.1007/s10397-016-0930-z (PMC4854942; doi:10.1007/s10397-016-0930-z)
Supplement: Supplementary file 1 — (PDF 191 kb) [file 10397_2016_930_MOESM1_ESM.pdf]

Journal of Gynecological Surgery

Laparoscopic versus robotic-assisted sacrocolpopexy for pelvic organ prolapse: a systematic review

Geertje Callewaert[1,2], Jan Bosteels[3], Susanne Housmans[2], Jasper Verguts[2,4], Ben Van Cleynenbreugel[1,5], Frank Van der Aa[1,5], Dirk De Ridder [1,5], Ignace Vergote[2,6], Jan Deprest[1,2]

1. KU Leuven, Department of Development and Regeneration, Cluster Organ Systems, Faculty of Medicine, Group Biomedical Sciences, 3000 Leuven, Belgium
2. Department of Obstetrics and Gynaecology, Division Woman and Child, University Hospitals Leuven, 3000 Leuven, Belgium
3. Belgian Center for Evidence Based Medicine (CEBAM), Belgian Branch of the Cochrane Collaboration, 3000 Leuven, Belgium
4. Department of Obstetrics and Gynaecology, Jessa Hospital, 3500 Hasselt, Belgium
5. Department of Urology, University Hospitals Leuven, Leuven, Belgium
6. Department of Gynaecologic Oncology, Leuven Cancer Institute, University Hospitals Leuven, KU Leuven, 3000 Leuven, Belgium

Corresponding author:

Jan A. Deprest, MD, PhD, FRCOG.

Department of Obstetrics and Gynaecology, UZ Leuven, Herestraat 49, 3000 Leuven, Belgium.

E-mail: jan.deprest@uzleuven.be

Tel.: +32 16 34 42 15

Fax: +32 16 34 42 05

Search strategy

SEARCH STRATEGY MEDLINE VIA PUBMED (> 1950 - 31.01.2015) - 93 records(validated and EndNote compatible)

(93 results)

((randomized controlled trial[pt] OR controlled clinical trial[pt] OR randomized[tiab] OR placebo[tiab] OR "drug therapy"[Subheading] OR randomly[tiab] OR trial[tiab] OR groups[tiab]) NOT ("animals"[MeSH Terms] NOT "humans"[MeSH Terms])) AND ((((((sacrocolpopexy[All Fields] AND "Laparoscopy"[Mesh]) OR (("laparoscopy"[MeSH Terms] OR "laparoscopy"[All Fields] OR "laparoscopic"[All Fields]) AND sacrocolpopexy[All Fields])) OR (("laparoscopy"[MeSH Terms] OR "laparoscopy"[All Fields] OR "laparoscopic"[All Fields]) AND ("sacrum"[MeSH Terms] OR "sacrum"[All Fields] OR "sacral"[All Fields]) AND colpopexy[All Fields])) OR (("laparoscopy"[MeSH Terms] OR "laparoscopy"[All Fields] OR "laparoscopic"[All Fields]) AND hysteropexy[All Fields])) OR (("robotics"[MeSH Terms] OR "robotics"[All Fields] OR "robotic"[All Fields]) AND sacrocolpopexy[All Fields])) OR (sacrocolpopexy[All Fields] AND (("robotics"[MeSH Terms] OR "robotics"[All Fields] OR "robotic"[All Fields]) AND versus[All Fields] AND ("laparoscopy"[MeSH Terms] OR "laparoscopy"[All Fields] OR "laparoscopic"[All Fields]))))

Last update: 31.01.2015

SEARCH STRATEGY EMBASE via EMBASE.COM (1974 -01.02.2015) - 119 records (validated and EndNote compatible)

SIGN filter for RCTs translated to EMBASE.COM

#1 'clinical trial'/exp
#2 'randomized controlled trial'/exp
#3 'randomization'/exp
#4 'single blind procedure'/exp
#5 'double blind procedure'/exp
#6 'crossover procedure'/exp
#7 'placebo'/exp
#8 randomi?ed AND controlled AND trial* AND [embase]/lim
#9 rct AND [embase]/lim
#10 'random allocation' AND [embase]/lim
#11 'randomly allocated' AND [embase]/lim
#12 'allocated randomly' AND [embase]/lim
#13 allocated NEAR/2 random AND [embase]/lim
#14 'single blind$' AND [embase]/lim
#15 'double blind$' AND [embase]/lim
#16 (treble OR triple) NEAR/2 blind$ AND [embase]/lim
#17 placebo$ AND [embase]/lim
#18 'prospective study'/exp
#19 #1 OR #2 OR #3 OR #4 OR #5 OR #6 OR #7 OR #8 OR #9 OR #10 OR #11 OR #12 OR #13 OR #14 OR #15 OR #16 OR #17 OR #18
#20 'case study'/exp
#21 'case report' AND [embase]/lim.
#22 'abstract report'/exp
#23 'letter'/exp
#24#20 OR #21 OR #22 OR #23
#25#19 NOT #24
#26'animal'/exp
#27'human'/exp
#28#26 NOT #27
#29#25 NOT #28
Latest update: 31.01.2015

SEARCH STRATEGY EMBASE via EMBASE.com (1974 - 01.02.2015) - 119 records
----
Database: Embase <1980 to 2015
Search Strategy:

#1 'laparoscopy'/exp OR 'laparoscopy' (113,732)
#2 sacrocolpopexy (1,098)
#3 #1 AND #2 (323)
#4 'laparoscopic'(120,332)
#5 #2 AND #4 (576)
#6 'sacrum' (9,862)
#7 'sacral' (17,563)
#8 #6 OR #7 (23,134)
#9 #1 OR #4 (150,301)
#10 #8 OR #9 (172,634)
#11 #2 AND #10 (682)
#12 hysteropexy (197)
#13 #9 AND #12 (78)
#14 'robotic sacrocolpopexy' (140)
#15 #3 OR #5 OR #11 OR #14 (746)
#16 'clinical trial'/exp (994,926)
#17 'randomized controlled trial'/exp (356,105)
#18 'randomization'/exp (64,127)
#19 'single blind procedure'/exp (19,082)
#20 'double blind procedure'/exp (118,740)
#21 'crossover procedure'/exp (41,025)
#22 'placebo'/exp (263,439)
#23 randomi?ed AND controlled AND trial* AND [embase]/lim (409,444)
#24 rct AND [embase]/lim (15,934)
#25 'random allocation' AND [embase]/lim (1,200)
#26 'randomly allocated' AND [embase]/lim (18,621)
#27 'allocated randomly' AND [embase]/lim (1,745)
#28 allocated NEAR/2 random AND [embase]/lim (714)
#29 'single blind$' AND [embase]/lim (21,395)
#30 'double blind$' AND [embase]/lim (167,309)
#31 (treble OR triple) NEAR/2 blind$ AND [embase]/lim (379)
#32 placebo$ AND [embase]/lim (316,284)
#33 'prospective study'/exp (268,740)
#34 #16 OR #17 OR #18 OR #19 OR #20 OR #21 OR #22 OR #23 OR #24 OR #25 OR #26 OR #27 OR #28 OR #29 OR #30 OR #31 OR #32 OR #33 (1,507,204)
#35 'case study'/exp (31,318)
#36 'case report' AND [embase]/lim (1,454,161)
#37 'abstract report'/exp (89,640)
#38 'letter'/exp (836,221)
#39 #35 OR #36 OR #37 OR #38 (2,278,819)
#40 #34 NOT #39 (1,454,452)
#41 'animal'/exp (19,822,732)
#42 'human'/exp (15,411,220)
#43 #41 NOT #42 (4,411,512)
#44 #40 NOT #43 (1,400,891)
#45 #15 AND #44 (119)

Last update: 01.02.2015

SEARCH STRATEGY COCHRANE: 24 records; last update 31/01/2015

Database: the Cochrane Library (all databases): Issue 1 of 12, January 2015

Search Strategy:

Search Name: Cochrane sacrocolpopexy

Last Saved:31/01/2015 18:03:24.343

Description:

IDSearch

#1MeSH descriptor: [Laparoscopy] explode all trees (4931)

#2sacrocolpopexy (70)

#3robotic (697)

#4hysteropexy (15)

#5#2 or #4 (83)

#6#1 or #3 (5499)

#7#5 and #6 (24)

Last update: 31.01.2015
